# Insight to Functional Conformation and Noncovalent Interactions of Protein-Protein Assembly Using MALDI Mass Spectrometry

**DOI:** 10.3390/molecules25214979

**Published:** 2020-10-28

**Authors:** Marco Giampà, Elvira Sgobba

**Affiliations:** 1 MR Cancer Group, Department of Clinical and Molecular Medicine, Norwegian University of Science and Technology, Olav Kyrres Gate 9, 7030 Trondheim, Norway; 2 Genetics and Plant Physiology, Department of Forest Genetics and Plant Physiology, Swedish University of Agricultural Sciences, 90183 Umeå, Sweden; elvira.sgobba@slu.se

**Keywords:** MALDI, protein assembly, noncovalent interactions

## Abstract

Noncovalent interactions are the keys to the structural organization of biomolecule e.g., proteins, glycans, lipids in the process of molecular recognition processes e.g., enzyme-substrate, antigen-antibody. Protein interactions lead to conformational changes, which dictate the functionality of that protein-protein complex. Besides biophysics techniques, noncovalent interaction and conformational dynamics, can be studied via mass spectrometry (MS), which represents a powerful tool, due to its low sample consumption, high sensitivity, and label-free sample. In this review, the focus will be placed on Matrix-Assisted Laser Desorption Ionization Mass Spectrometry (MALDI-MS) and its role in the analysis of protein-protein noncovalent assemblies exploring the relationship within noncovalent interaction, conformation, and biological function.

## 1. Introduction

**Figure 1:**
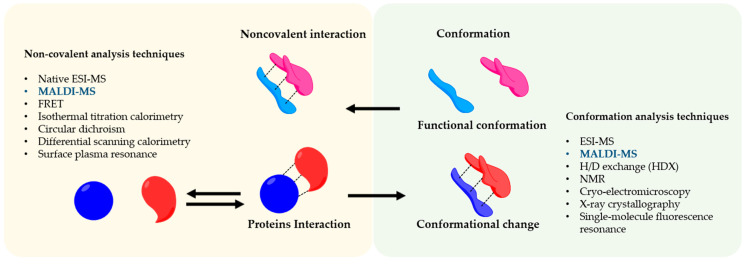
Noncovalent Protein-protein assembly. The picture shows noncovalent interactions in protein-protein complexes and an overview of the techniques to study their conformation and interaction.

Matrix-assisted laser desorption ionization mass spectrometry (MALDI-MS) was the first time developed by Hillekanmp and Karas [1]. Briefly, the irradiation of a laser with a specific wavelength on a solid sample, covered with a small UV absorbing molecule, leads to the production of gaseous ions, that are analyzed based on their mass-to-charge ratio (*m*/*z*) by a mass spectrometer [2]. This technique is applied to several scientific fields from chemistry to biomedicine because can detect a broad variety of compounds such as small molecules until large proteins using a very small amount of sample. MALDI-MS is commonly used in proteomics to identify proteins and their post-translational modifications by detection of tryptic peptides and its comparison to specific databases [3,4]. On the other hand, the soft nature of the MALDI allows also us to analyze entire protein complexes that are bound by noncovalent interactions [5]. Understanding noncovalent interactions between proteins are of great interest and crucial for the biological function such as enzymatic catalysis [6], modulation of signal transduction [7], and immunomodulation [8]. In this regard, the conformation of the protein has a strong effect on the noncovalent interactions and therefore on biological effects [9]. The information regarding the influence of the conformation in the interaction and vice versa is a great interest not only for basic research or structural biology but also in the pharmaceutical fields in which protein-based drugs are continuously developed e.g., vaccines [10]. The interaction of antigen and antibody is a great example of how the conformation of the antigen is crucial for its interaction [11,12]. Therefore, noncovalent interactions are involved in the functionality of a complex protein network [13]. To analyze this complex and sensitive information, sophisticated methods are applied to preserve the conformational and reactivity of the protein, as shown in Figure 1.

In this scenario, mass spectrometry with its high sensitivity, resolution, and accuracy gives a strong contribution to understanding the chemical reasons for a noncovalent interaction within proteins [14].

In this review, the principle of noncovalent interactions, protein folding/conformation, and their biological function is explained, with the structural proteomics. Therefore, MALDI mass spectrometry will be introduced as an analytically relevant contribution to understanding functional conformation and specific noncovalent interactions.

## 2. Functional Conformations and Noncovalent Interactions

The amino acid sequence of a protein dictates the conformational folding to which it undergoes determining secondary, tertiary, and ultimately quaternary structure. Protein folding is a process in which a highly dynamic equilibrium between folded and unfolded states, dictated by its covalent backbone and by noncovalent interactions between amino acid side chains, determines its native three-dimensional structure, also called conformation [15]. In the case of proteins with more polypeptides chain, each of that constitutes a protein subunit e.g., homomers or hetero-oligomers in which identical or different subunits are interacting respectively [16].

Homomers can form intertwined assemblies, whereby small fragments of the protein called domains are swapped between two interacting subunits [17]. This results in different types of quaternary arrangements, ranging from dimers to polymer, reviewed broadly by Wodak et al. [18].

It has been shown that homodimers are involved in allosteric regulation, whereby a ligand binding event at one site of the protein induces conformational changes affecting the binding activity elsewhere in the protein [19,20].

**Figure 2:**
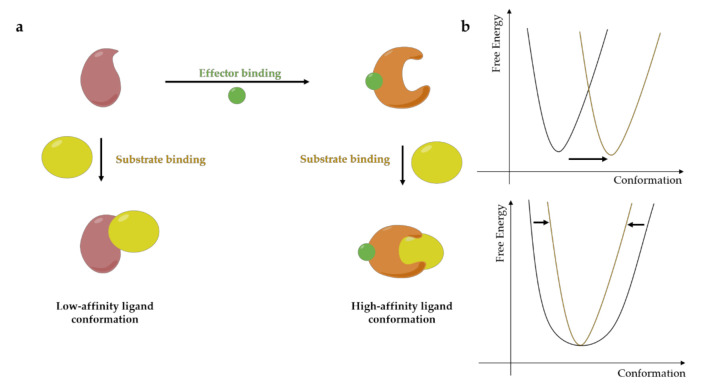
Allosteric binding and it conformational dynamics in (**a**) effector binding at the allosteric site lead to substrate binding at the active site. (**b**) Allosteric binding may result in a conformational change, resulting in a movement of the corresponding free energy basin from one region to another region in conformational space, or the broadness of the free energy basin (adapted from [21]).

Allosteric regulation is an important feature that can regulate the function of a protein in a positive (effector) or negative (inhibitor) manner through conformational changes. As an example, the binding of the effector, that can be a small molecule or a protein, at a distal site from the active site can change its ligand affinity at the active site due to conformational changes post-binding of the effector [21,22]. Consequently, spatial conformational changes in the basin of free energy are observed as schematically reported in Figure 2.

Allosteric regulation has been primarily observed in enzymes involved in branching points of a metabolic pathway, in receptors sensible to mild signal perturbation [23] as well as in polymeric proteins, whereby this type of regulation can act in a change of activity arising from changes in subunit–subunit contacts [24].

The process of generally homo-oligomer formation can contribute to enhancing protein stability, protecting against aggregation in some systems [25]. The process of oligomerization is regulated by different mechanisms: 1) binding of molecules at the oligomerization interface, resulting in a blockage of the process; 2) binding of a molecule on a distal area of the oligomerization interface resulting in a stabilization of the interacting oligomers instead. Additionally, as described by Jaffe et al. a protein can exist in lower oligomeric structures, called morpheeins, which dictates a defined stoichiometry for the higher-order oligomeric structure [26]. The transition between the oligomeric states requires dissociation of the oligomer and a change in conformation before the other oligomeric state is formed. Therefore, molecules that allosterically stabilize a certain conformation of the lower oligomeric structure will shift the oligomerization equilibrium towards the corresponding higher oligomeric one [27].

An example is the equilibrium between high active octamers and low inactive hexamers of the enzyme porphobilinogen synthase (PBGS) involved in cellular respiration [26]. PBGS is allosterically activated by the binding of magnesium at the interface which stabilizes the high active octamers [26], whereas the low active examers are stabilized by the inhibitor morphlock-1 [26].

A similar mechanism is observed in tumor factor p53, which explicates its function and activity as a tetramer. The process of tetramerization of the p53 tumor factor is essential for its tight binding to DNA elements and ultimately to induce transcription of p53 target genes. Its equilibrium is regulated by interactions with other proteins, such as proteins from the 14-3-3 and S100 families and numerous kinases [28].

In the case of protein interacting with other proteins or molecules, allosteric cooperativity can play a major role not only in the protein folding but as well in the binding receptor-ligand, whereby their function is ligand concentration-dependent [29,30]. This is the case of the binding of O_2_ to hemoglobin as well as in allosteric enzymes [31] e.g., ribonucleotide reductases involved in the *de novo* DNA biosynthesis; the aspartate transcarbamoylases involved in pyrimidine biosynthesis, in which cytidine triphosphate (CTP) acts as an allosteric inhibitor, and adenosine triphosphate (ATP) functions as an allosteric activator [32].

At last, the recognition process between a membrane receptor and a pharmacological agent, resulting ultimately in allosteric activation of ligand-gated ion channels and G-protein-coupled receptors [33].

At the basis of such allosteric cooperativity, there are noncovalent interactions of proteins that are essential in different biological processes, e.g., signaling cascades, enzyme-substrate, or enzyme-drug interactions, and multi-protein types of machinery.

Therefore, proteins are dynamic structures with a defined temporal and spatial conformational flexibility, empowering them to perform specific functions. The interconversion of the protein from one conformation to another is associated with specific free energy [34]. It allows ultimately the protein to function, e.g., a protein requires conformational changes to exploit functions such as catalysis, ligand binding, or signal transduction [35]. The understanding of protein dynamics can give us a picture of the protein function and the biological process [36].

Protein residues are strictly involved in its dynamic structure and strongly related to its function [37], e.g., the opening and closing of the apo maltose-binding protein domains [38].

At the basis of such tertiary and quaternary protein structure, there are noncovalent interactions, which are involved as well in other biomolecules complexes, e.g., proteins, DNA, RNA, carbohydrates. The main noncovalent interactions are hydrogen bonds, ionic bonds, hydrophobic interactions, Van der Waals force, and π-π interactions [16]; they are weaker than covalent ones as well explained in Israelachvili et al. [39]. They are involved in biological processes (protein-protein interactions, protein-small ligands, protein-DNA, DNA-DNA, DNA/RNA-small ligands). In Table 1, a list of noncovalent protein interactions with their respective bond energy is reported.

Electrostatic forces are strong determinants of disordered protein ensembles. The relationship between electrostatics, conformational ensembles, and quaternary interactions is unclear. Therefore, the impact of electrostatic interactions on the association of calmodulin and calcineurin phosphatase was explored and showed how charged amino acids could affect the conformation ensembles and the binding rate between the two proteins [40].

This interaction usually involves the hydrophobic side groups of the amino acid contributing the carboxyl group to the bond [39].

The interaction energies (Δ*E*_gas_) of a noncovalent interaction can be estimated by subtracting the calculated energies of the interacting monomers (*E*_monomer1_ and *E*_monomer2_) from the total energy of the complex (*E*_complex_) according to Equation (1).

Δ*E*_gas_ = *E*_complex_ – *E*_monomer1_ – *E*_monomer2_ (1)

Since noncovalent interactions are involved in different biological processes, their study is essential. Mass spectrometry (MS) has a unique ability to preserve weaker noncovalent bonds when transferring species from solution into the gas phase. In fact, MS has been proven to be extremely useful to study and characterize protein conformations in noncovalent protein complexes, including their dynamic behavior. This review will address protein-protein complexes, given their ubiquity in biology processes [41].

## 3. Types of Protein and Peptide Noncovalent Self-Assembly and Their Biological Function

Nature provides good examples of self-assemblies, e.g., the phospholipid bilayer or microtubules involved in the cytoskeleton of the mitotic spindle in prokaryotes and eukaryotes cell division [42]. Typical self-assembly structures include micelles, vesicles, and fibrillar structures (nanotubes, fibers, and amphiphilic peptides [43]. Although self-assembly occurs in an aqueous environment, water molecules are not involved in the establishment of intermolecular noncovalent interactions during the formation of assembled structures [42]. If on one side weakness of noncovalent interactions contribute to stabilizing self-assembly, on the other side, make self-assembly vulnerable toward temperature, pH, and concentration [44]. Such a type of self-assembly occurs within peptide chains (5–10 aa), as well as between protein subunits, defining its quaternary structure. The latter can be involved as well in assembly with other proteins, e.g., assembly ligand-receptor. For these, the microenvironment around the protein is crucial to the self-assembly of proteins/peptides because it alters the tertiary and quaternary structures of the protein that are closely correlated to the chemistry of noncovalent interactions. For example, hydrogen bonding between the backbone of peptide chains, although it contains different amino acid sequences, is an essential factor in peptide self-assembly, as it drives longitudinal packing of peptide monomers into β-sheets. On the other hand, inter β-sheet interactions among side chains of hydrophilic and hydrophobic residues of peptide molecules, regulates the lateral packing and the final assembled morphology [45]. Therefore, the combination of the primary structure of the protein effect the quaternary structure of self-assembled proteins.

### 3.1. Peptide Self-Assembly

Generally, amphiphilic peptides are self-assembly characterized by two functional groups: A hydrophobic tail group, usually an alkyl chain, involved in hydrophobic interactions; a functional groups able to establish intermolecular hydrogen bonds responsible for the curvature of the self-assembly [46]. Therefore, the driving force of the self-assembly is the amphiphilicity. Although the driving force of the self-assembly is the amphiphilicity, exposing the functional groups on the surface [43], hydrophobic regions are beneficial especially in drug delivery; an example is given by tandem dimers of peptides harboring binding sites for LDL (low-density lipoprotein) receptor and cell-surface heparin sulfate proteoglycans [42]. Therefore, amphiphilic peptides, because of their dual nature hydrophilic and hydrophobic, are primarily used for different applications, e.g., drug delivery, cell trafficking, tissue engineering, and antimicrobial agents [43].

Peptide self-assemblies include lipopeptides e.g., the antimicrobial agent daptomycin [47]; surfactant- like peptides (SLP) e.g., TAT (YGRKKRRQRRR), an arginine-rich peptide from HIV retrovirus [48]; cationic peptides e.g., surfactin [49]; hydrogels e.g., amyloid fibrils FMOC-arginine-glycine-aspartate [50,51] and peptoids which are abundantly used for biomedical purposes [42].

Whether there is a relationship between bioactivity and peptide self-assembly is still an open question. Nevertheless, they continue to gain attention due to their biocompatibility, biodegradability, and bio functionality.

### 3.2. Protein Self- and Non-Self-Assembly

Protein-protein self-assemblies are involved in numerous amounts of biological processes, where they present a homo-oligomeric structure in which identical protein subunits associate. The process of oligomerization can regulate the function of ion channels, receptors, transcription factors, and enzymes, but can lead to the pathogenic condition when nonnative oligomers are associated [52].

In the case of enzymes, oligomerization plays an essential role in enzyme regulation, e.g., enzyme activation or deactivation based on enzyme concentration. Alternatively, oligomerization can regulate an enzyme allosterically by promoting cofactors binding to a non-substrate site, facilitating substate-induced cooperativity [52].

Protein-protein self-assemblies include proteins involved in molecules transport e.g., hemoglobin [31]; aquaporin CLIC1 [53], transferrin, and Tat protein [54] involved in O_2_, water, chloride ions, iron, and folded protein respectively. Additionally, oligomerization can participate in the transferring of signals across the cell membrane, as a response to the recognition of an agonist by the receptor, enzyme activation. An example of receptor activation is given by G-protein coupled receptors [55], protein G [56], TNF, and TNFR [57]. Oligomerization can be found as well in detoxifying enzymes eg. dimers of human glutathione S-transferase A1-1 (hGST), which forms a noncovalent adduct with one molecule of glutathione (GSH), and two molecules of water [58].

A biologically and medical-relevant example of protein assembly is the one established between antigen-antibody, which results in an immune response e.g., the assembly of MHC, major histocompatibility complex with epitope regions of the antigen [59,60,61,62].

The process of oligomerization can be observed intracellularly e.g., the noncovalent complex given by post-translational modifications of ubiquitin-like proteins, which require the aid of three enzymes (E1, E2, E3) [63]; and the electron transfer complexes cytochrome *c*‒cytochrome *c* peroxidase and amicyanin‒methylamine dehydrogenase‒cytochrome c_551i_ [64].

Those examples show the strong interdependency between conformation and function; therefore, the understanding of protein-protein noncovalent complexes might help to elucidate the functional mechanism and help in engineering noncovalent assemblies as potential drugs or drug carriers.

## 4. Techniques to Understand Protein Assembly Interactions and Conformations

The determination of a protein is studied at different levels by implementing a wild set of experimental techniques that provide a set of information regarding the three-dimensional structure, the conformation of a protein along a specific environment.

Briefly, the X-ray crystallography reveals static three-dimensional structure at an atomic resolution [65]; NMR spectroscopy can study the structure and the conformational dynamics of proteins in solution on its native-like/physiological environment [66,67,68]; circular dichroism can monitor structural changes of proteins [69] and recently it is combined with mass spectrometry [70]; cryo-electro microscopy (cryo-EM) allows to observe structures in their near-native environment and with a near-atomic resolution [71].

The most important challenge of these techniques is the conformational analysis of intrinsically disordered protein (IDP) and its protein-protein interactions due to its rapid interconversion within several conformations or so-called conformational heterogeneity [72,73,74].

Therefore, single-molecule fluorescence resonance, sensitive to transient conformational populations, can provide the time resolution to snap different conformations and the dynamic of protein interactions [75]. Other techniques that provide information about protein-protein interaction are isothermal titration calorimetry [76], differential scanning calorimetry [77], and surface plasmon resonance [78].

The conformational data elaborate by those techniques provides a conclusive picture of the relationship between structure and protein function. For this purpose, several bioinformatics tools such as molecular dynamics (MD) were proposed to unravel the information hidden in conformational ensemble data and to guide toward the rationalization for the data [79].

## 5. MALDI Mass Spectrometry Analysis as an Approach to Unravel Interactions in Protein Assembly Guided by Molecular Conformation

**Figure 3:**
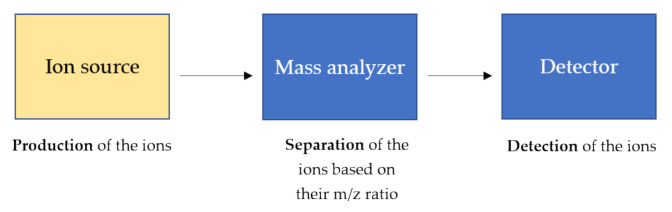
General components of a mass spectrometer. The ions, produced by the ion source, are separated based on their *m*/*z* ratio and detected by high sensitive detectors. The ion source produces the ions in the gas phase from the sample. In the mass analyzer, such as triple quadrupole and time-of-flight (TOF), the ions are separated based on the *m*/*z* charge ratio and at the end, the ionic currents are detected by conversion to electrical signals in the detector.

Mass spectrometry is a technique able to measure the *m*/*z* of several ionized compounds that are analyzed through the use of a mass analyzer and highly sensitive detectors giving several advantages in analytical procedures such as high specificity, sensitivity, speed, and stoichiometry [80,81]. In general, a mass spectrometer consists of three components as schematically shown in Figure 3.

The ion source is the main component and the heart of the mass spectrometer in which the analytes are ionized through specific and diverse ionization processes, and therefore detectable by the mass spectrometer. The parameters of the ion source and the sample preparation strongly affect the energy stability of noncovalent interactions, that can be preserved during the ionization process in the gas phase [82]. Therefore, the ion source and its ionization process strongly affect the sensitivity of the noncovalent structures such as the tertiary and quaternary structures of proteins.

Soft ionization processes, such as electrospray ionization (ESI) and MALDI, are employed for structural analysis of the protein elucidating not only noncovalent interactions but also conformational changes of the proteins. For such purpose, several techniques such as hydrogen/deuterium exchange (HDX), native MS, limited proteolysis, cross-linking, and fast photochemical derivatizations are applied to investigate the relationship function and conformation [16,83].

**Figure 4:**
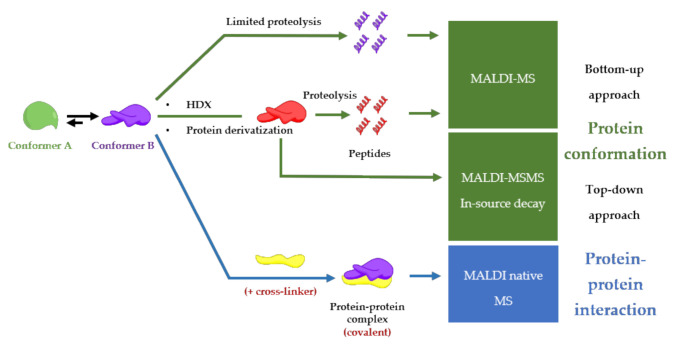
Conformation and protein-protein interaction study based on MALDI-mass spectrometry finding the functional conformation.

Although Electrospray Ionization (ESI) is the preferred method to study conformations and noncovalent interactions between proteins [84], MALDI-MS remains a simple and fast alternative to study protein-protein complexes. Even though the high energy of the laser, MALDI can preserve noncovalent interactions [5], and yields stoichiometric information of the protein-protein complex [16]. A general advantage of implementing MALDI in analytical procedures is also a user-friendly sample preparation (small sample volumes, no complicated protocols, and high salt tolerance), compatible with high-throughput screening procedures [85] and clinical applications [86]. On the other hand, special and expensive detectors such as ion conversion dynode (ICD), which is the only commercially available high-mass detector, are required for the detection of intact protein-protein complexes with very good sensitivity [87]. Another disadvantage of MALDI-MS to take into account is the challenging in the performance of absolute quantification due to sample composition, sample morphology, inhomogeneous matrix crystallization, laser conditions and, depletions during laser exposure [88]. This review is going to describe MALDI-MS-based procedures for finding functional conformations of a protein involved in a protein-protein complex as described in Figure 4 and all the examples are summarized in Table S1.

### 5.1. Limited Proteolysis MALDI-MS

Limited proteolysis mass spectrometry is a trend method to study conformational effects upon interaction with small molecules [89]. The limited proteolysis is restricted digestion of the protein with a low concentration of proteases. It leads to controlled proteolysis at putative cleavage sites, based on the backbone plasticity and/or local unfolding of the protein region [90].

**Figure 5:**
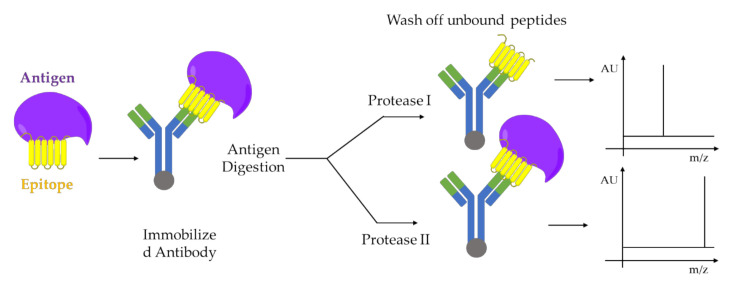
Epitope analysis by limited proteolysis-MALDI-MS approach. Two proteases I and II, which cleaves the antigen at different sites, are used for digestion. The immobilized antibody-epitope complex can be analyzed directly by MALDI-MS. Adapted from [100].

A combination of limited proteolysis and MALDI-MS was applied for the first time to study a multi-component biomolecular assembly DNA-protein [91] and to perform structural studies using proteolytic footprinting [92]. Similar approaches were applied to protein-protein interactions between cell cycle regulatory proteins, p21 and Cdk2 [93,94], or on capsid viral protein complexes to elucidate mobile features of the viral proteins [95] e.g., autocatalytically cleavable protein α in Flock House virus [96]. The activation-dependent conformational changes of ß-arrestins are revealed using limited proteolysis in combination with both sodium dodecyl sulfate - polyacrylamide gel electrophoresis (SDS-PAGE) and mass spectrometry analysis. This mass spectrometry-based method can be adapted as a tool to study the nature of ß-arrestins conformational changes downstream of different receptors, as well as to study how each conformational change can be associate with a specific ß-arrestin function e.g., desensitization, internalization, and signaling [97,98]. The structure-function dependent interaction between Hsp90 and its co-chaperone Cdc37 resulting in the facilitation of folding and activation of numerous proteins. Such interaction was studied by limited proteolysis in conjunction with MALDI-TOF-MS analysis of peptide fragments and peptide microsequencing [99]. Another example is given by epitope analysis of antibody-antigen complexes performed also by limited proteolysis coupled with MALDI-MS [100] as shown the Figure 5.

### 5.2. HDX-MALDI-MS

HDX is a powerful labeling tool that investigates protein conformational alteration by monitoring differential deuterium incorporation in altered regions [101]. Protein hydrogens attached to electronegative atoms (e.g., amide hydrogens) continuously exchange with solvent hydrogen and deuterium either in an acid or base-catalyzed manner [102,103]. The measurement of the exchange rate for these amides provides detailed information about their surrounding environments [104,105]. In HDX-MS there are two ways to measure the exchange rate of the amides: 1) by measure the m/z ratio of the peptides produced after proteolysis of deuterated protein (“bottom-up approach”) and 2) by measure the m/z ratio of fragments produced by ion dissociation, for example, electron-capture dissociation (ECD) or electron-transfer dissociation (ETD), of the deuterated protein avoiding randomization of deuterons within proteins in the gas phase (“top-down approach”) [101]. An interesting application of HDX coupled with mass spectrometry and NMR spectroscopy consists of studying the conformational and dynamic changes of an allosteric system, consisting of a transition of the system within several conformational states [106]. HDX-MShas enabled the investigation of protein folding intermediates, the characterization of transient structure in intrinsically disordered protein [107].

An example of the bottom-up approach was reported by Mandel et al., where the combination of HDX-MALDI-MS was employed to identify peptic fragments from protein complexes at the interacting interface between kinase inhibitors and cyclic-AMP-dependent protein kinase. This complex retains deuterium under hydrogen exchange conditions due to decreased solvent accessibility at the interface of the complex [108,109]. The isotope exchange of polypeptide backbone amide hydrogens of hemoglobin (Hb) and following tryptic digestion was carried out inside red blood cells and monitored using MALDI-MS to explore the conformational transition associated with oxygenation [110]. Conformational dynamics of factor XIII activation induced by calcium were also investigated by HDX-MALDI-MS of tryptic peptides [111]. In this context, limited proteolysis combined with HDX and quantitative LC-MALDI-MS was employed to monitor the conformational changes of troponin C after binding with ligand calcium [112].

On the other hand, a HDX-MALDI-MS using a top-down approach trough in-source decay analysis of deuterated cytochrome c yielded an extensive series of c-fragment ions that originate from the cleavage of nearly all N-C_α_ bonds allowing for a detailed analysis of the deuterium content of the backbone amides with a minimal hydrogen scrambling. In this way, the dynamic behavior of cytochrome c in solution is accurately reflected in the deuterium content of the fragment ions [113].

Moreover differential and native HDX-MALDI-MS semiautomated conformational screening was performed by changing the polarity of the solvent and different conformations of the protein were evaluated [114]. In detail, the small size proteins, ubiquitin, and insulin were analyzed by using a nonaqueous matrix solution (5 mg/mL of sinapic acid in acetonitrile with 0.1% 3-nitrobenzyl alcohol) to minimize the D/H back exchange. Spatially resolved deuteration levels are readily obtained by mass analysis of consecutive fragment ions. This semi-automated approach is an important step forward in the automation of proteomics approaches that are ongoing developing for clinical and biomedical applications [115].

The coupling of MALDI-MS to HDX is limited by undesired back-exchange reactions from the air humidity, but optimized protocols were developed to minimize this issue in sample preparation [116]. For example, the low temperature of the sample plate minimizes the amount of back exchange [109].

### 5.3. Protein Derivatization

Since chemical modification has a conformational impact on protein, protein derivatization is another approach to study the functional conformation of a protein [117,118]. The yield of these derivatizations depends on the spatial localization of amino acids in the protein and therefore on the protein folding. In MALDI-MS, the bottom-up approach of derivatized protein is employed for conformational studies and native MS. The top-down approach for the conformational study is still carried out with ESI coupled mass spectrometers in which infrared multiple photon dissociation (IRMPD) and electron capture/electron transfer dissociation (ECD/ETD) are employed to dissociate the derivatized protein and to analyze the cross-linked fragments. On the other hand, through in-source decay, a top-down approach can be carried out by MALDI-MS, but so far, an established method is not reported with derivatized protein. Moreover, the protein derivatization is employed to convert noncovalent protein-protein complex, such as integral membrane protein complexes, to covalent, and therefore the resulting covalent complex is detected by MALDI-MS [119]. One of the chemical modifications mostly used in MALDI-MS is cross-linking (CL). The concept of protein cross-linking in combination with mass spectrometry holds great promise to derive structural information on protein conformation, protein-protein interactions, and protein interaction networks in complex biological samples [120,121]. The general workflow based on MALDI is the detection of peptide derivates from cross-linked proteins with a specific conformation and/or specific interaction with another protein using MALDI-MS/MS and homo-bifunctional amino group-specific [122] and isotope-labeled cross-linkers [123].

Hundreds of chemicals described in the literature or commercially available are based on a limited number of reactions [124,125]. Examples of cross-linkers used in MALDI-MS are amino-reactive cross-linkers (*N*-hydroxysuccinimide esters) [126], sulfhydryl-reactive cross-linkers, maleic acid imides, disuccinimidyl suberate, bis(sulfosuccinimidyl suberate and 1,1′-(suberoyldioxy) bis azabenzotriazole [127], glutaraldehyde [119,128] and photo-reactive cross-linkers [129,130,131].

Promising for future applications in structural proteomics and imaging is the development of a derivatization method based on nanosecond photoreactions of neuropeptides using nitrobenzaldehyde giving the reactive 2-nitrosobenzoic anion able to react with the primary group of a protein [132]. By combining the use of bisthiopropionic acid *N*-succinimidyl ester (BipS), a photocleavable, isotopically coded, fluorescent cross-linker, the first direct evidence was provided for the docking site of a phosphorylated G-protein-coupled receptor C terminus on the multifunctional adaptor protein beta-arrestin, clearly demonstrating the broad potential and application in structural and cellular biology [133]

### 5.4. MALDI Native Mass Spectrometry

First studies reported that the observation of entire noncovalent complexes, without using any derivatization procedures, in high mass MALDI-MS is possible only by acquiring the mass spectra by the first few laser shots at a new sample spot (“first shot phenomenon”) [134,135,136].

Recently, Beaufour et al. developed a method for native mass spectrometry of proteinsbased on liquid deposits. The molecular arrangement between matrix and complex is involved in the stability of noncovalent interaction during the MALDI events. In contrast to the solid deposit that has an irreversible frozen arrangement, in liquid deposit, regeneration of an ideal rearrangement following a single-shot accumulation is hypothesized. Moreover, the gas phase dissociation was minimized by parametric optimization [137]. Therefore, the possibility to observe native protein complexes brings an important outlook for the newly established MALDI-MS imaging that leads native proteomics directly to a tissue specimen contributing to the histopathological medical diagnosis field [138]. The “native-MSI” methodology was further improved by using liquid extraction surface analysis in order to spatially resolve and identify intact proteins with a molecular weight up to 47 kDa, including trimeric protein complex, directly on rat kidney tissue section [139].

## 6. Conclusions

In conclusion, the interdependency between the conformation and the noncovalent interaction is an essential factor to understand and predict the protein behavior in a biological context. This investigation is relevant in the pharmaceutical, clinical, and structural biology fields, which finds application in engineering new drug candidates, new drug delivery systems, and tissue engineering. Therefore, MALDI-MS can infer the structural features of a protein and/or protein complex from a small volume of matrix co-crystallized sample. This allows us to perform high-throughput analysis of several proteins at different conditions of substrate, ligands, and co-factors. Especially, in pandemic time, the need for a fast-response tool to validate specific vaccines can be achieved with MALDI-MS, based on its previous applicability in studies of epitope-antibody interaction analysis. Furthermore, MALDI-mass spectrometry imaging can provide functional and spatial information of protein complex as a part of tissue In this regard, the intracellular spatial resolution in mass spectrometry imaging is developing [140,141,142] and it would be an advance to localize intracellular proteic conformers involved for example in tissue homeostasis where protein conformation controls the organization and dynamics of cell-matrix adhesion [143] as a step forward to a better understanding of tissue chemistry.

## Figures and Tables

**Table 1:** Types of noncovalent protein interactions and their bond energy compared to a covalent bond [39].

Name	Basis of Interaction	Bond Energy (kcal/mol)
Covalent bond	Sharing electron pairs	50–110
Hydrogen bond	Sharing H atom	3–7
Ionic bond	The attraction of opposite charges	3–7
Hydrophobic interaction	Interaction of non-polar group	1–2
Van der Waals interaction	Interaction of electrons in the presence of polar substances	1
π-π interaction	Π-molecular orbital interaction	2

## Supplementary Materials

The following are available online, Table S1: Protein complex and conformation studies using MALDI-MS based approaches.
