# Supplementary material for: Insight to Functional Conformation and Noncovalent Interactions of Protein-Protein Assembly Using MALDI Mass Spectrometry
Source: Molecules. 2020 Oct 28;25(21):4979. doi: 10.3390/molecules25214979 (PMC7662314; doi:10.3390/molecules25214979)
Supplement: Supplementary file 1 [file molecules-25-04979-s001.pdf]

Type of the Paper (Review)

Insight to functional conformation and noncovalent interactions of protein-protein assembly using MALDI mass spectrometry

Marco Giampà [1]* and Elvira Sgobba [2]

[1] Department of Clinical and Molecular Medicine, Norwegian University of Science and Technology, Olav Kyrres gate 9, 7030 Trondheim, Norway; marco.giampa@ntnu.no
[2] Department of Forest Genetics and Plant Physiology, SLU, Skogsmarksgränd 17, 90183 Umeå, Sweden; elvira.sgobba@slu.se
* Correspondence: marco.giampa@ntnu.no

Received: date; Accepted: date; Published: date

Supplementary Table 11 Protein complex and conformation studies using MALDI-MS based approaches

Protein complex	Type of analysis	Biological function	Matrix	Mass analyzer	Polarity	Reference
Molecular assembly DNA-protein	Limited proteolysis	Transcription factor important for regulation of cell development and proliferation	CHCA[1] (saturated of 1:3:2 (v/v) formic acid: water:isopropanol)	TOF[8]	+	[92]
HIV-1$_{sf2}$ P24 with anti-p24 antibody	Limited proteolysis (proteolytic footprinting)	Immune response to capsid protein p24 of HIV	CHCA[1] (saturated solution of 45:45:10 ethanol:water:formic acid)	TOF[8]	+	[93]
p21 and Cdk2	Limited proteolysis	p21 inhibits the activity of cyclin-CDK2 which regulates cell cycle progression at G1 and S phase	CHCA[1] (saturated solution with 1:3:2 (v/v) formic acid: water:isopropanol)	TOF[8]	+	[94]
Viral capsids	Limited proteolysis	Protein shell enclosing genetic material	SA[2] (saturated solution of acetonitrile/water (50:50) 0.25% TFA)	TOF[8]	+	[96,141]

Protein complex	Type of analysis	Biological function	Matrix	Mass analyzer	Polarity	Reference
β-Arrestin 2 - V2R-pp	Limited proteolysis	Intracellular signal transduction via GPCR receptor V2R-pp	SA[2] (saturated solution of 45% acetonitrile 0.1% TFA[3])	TOF[8]	+	[98,99]
Conformation cytocrome c	HDX	Cell apoptosis	SA[2] (20 mg/mL in 100% acetone (precoating))	TOF[8]/ISD[9]	+	[114]
Kinase inhibitor and cyclic-AMP-dependent protein kinase	HDX	Regulation of glycogen, sugar, and lipid metabolism	CHCA[1] (5 mg/ml in 1:1:1 acetonitrile, ethanol, and 0.1% TFA - pH adjusted to 2.5 with 2% of TFA)	TOF[8]	+	[109,110]
Ubiquitin and insulin	HDX native	E3 ubiquitin ligases directly degrades the insulin receptor, promoting insulin resistance	SA[2] (5 mg/mL in acetonitrile with 0.1% 3-nitrobenzyl alcohol)	TOF[8]	+	[115]
Hemoglobin - O_2	HDX	O_2 transport in the blood from lungs or gills to the rest of the body	CHCA[1] (5 mg/mL in 2:8:1 acetonitrile/ethanol/0.1% aqueous TFA)	Triwave™ Technology	+	[111]
Factor XIII activation with calcium	HDX	Thrombin and Ca^{2+} activate Factor XII in the process of blood coagulation	CHCA[1] (10 mg/ml in 1:1:1 ethanol/acetonitrile/0.1%TFA at pH 2.2)	TOF[8]	+	[112]

Protein complex	Type of analysis	Biological function	Matrix	Mass analyzer	Polarity	Reference
Troponin C with Ca^{2+}	Limited proteolysis, HDX and LC-MS	Muscle contraction	CHCA[1] (saturated solution of in 50% acetonitrile/49.5% water/0.5% TFA)	TOF[8]	+	[113]
Integral membrane proteins complex: BtuCDF	Cross-linking	Uptake of vitamin B_{12} in *E. coli*	SA[2] (20 mg/mL in water/acetonitrile /TFA, 49.95/49.95/0.1, (v/v/v))	TOF[8]	+ / -	[120]
Nanobody•Membrane Protein Complexes	Cross-linking	Chaperones in crystallization and blockers or modifiers of protein activity	SA[2] (10 mg/mL in acetonitrile/water /TFA, 49.95/49.95/0.1, (v/v/v))	TOF[8]	+	[129]
Covalent 14-3-3ζWT Homodimeric Product	Cross-linking (bottom-up approach)	Phosphorylation process	CHCA[1] (5 mg/mL dissolved in 50% (v/v) aqueous acetonitrile containing 0.1% (v/v) TFA)	TOF[8]	+	[132]
calmodulin and peptide M13 complex	Cross-linking	Calmodulin regulates calcium levels in the cell. M13 resembles skeletal muscle myosin light chain kinase (skMLCK).	DHB[4] (saturated matrix solution of 30% acetonitrile, 69.9% (water, 0.1% TFA)	TOF[8]	+	[131]
Trimeric porin	Native MS	Transport of hydrophilic molecules	FA[5] (20-50 mg/mL in pure THF)	TOF[8]	+	[135]

Protein complex	Type of analysis	Biological function	Matrix	Mass analyzer	Polarity	Reference
Tetramer streptavidin	Native MS	Biotin binding protein. As antibody-streptavidin complex	SA[2] (in water with 20 mM TEAB at pH 8.5 or FA in pure THF)	TOF[8]	+	[137]
HU$\alpha\beta$ protein (heterodimer)	Native MS	Regulation of cell transcription	Ionic liquid matrices	TOF[8]	+/-	[138]

1. α-cyano-4-hydroxycinnamic acid; 2. Sinapinic acid; 3. Trifluoroacetic acid; 4. 2,5-dihydroxybenzoic acid; 5. Ferulic acid 6. Tetrahydrofuran; 7. 5-methoxysalicylic acid; 7. triethylammonium hydrogen carbonate; 8. Time-of-Flight; 9. In-source decay

References

92. Cohen, S.L.; Ferré-D'Amaré, A.R.; Burley, S.K.; Chait, B.T. Probing the solution structure of the DNA-binding protein Max by a combination of proteolysis and mass spectrometry. *Protein Sci.* **1995**, *4*, 1088-1099, doi:10.1002/pro.5560040607.
93. Tomer, K.B.; Trojak, S.J.; Parker, C.E. Structural Studies of Protein-Protein Interactions Using Proteolytic Footprinting and MALDI/MS. In *New Methods for the Study of Biomolecular Complexes*, Ens, W., Standing, K.G., Chernushevich, I.V., Eds. Springer Netherlands: Dordrecht, 1998; 10.1007/978-94-015-9046-4_4pp. 59-65.
94. Kriwacki, R.W.; Wu, J.; Siuzdak, G.; Wright, P.E. Probing Protein/Protein Interactions with Mass Spectrometry and Isotopic Labeling: Analysis of the p21/Cdk2 Complex. *JACS* **1996**, *118*, 5320-5321, doi:10.1021/ja960752m.
96. Siuzdak, G. Probing viruses with mass spectrometry. *J. Mass Spectrom.* **1998**, *33*, 203-211, doi:10.1002/(sici)1096-9888(199803)33:3<203::Aid-jms653>3.0.Co;2-q.
98. Zhao, Y.; Xiao, K. A Mass Spectrometry-Based Structural Assay for Activation-Dependent Conformational Changes in β-Arrestins. In *Beta-Arrestins*, Springer: 2019; pp. 293-308.
99. Xiao, K.; Shenoy, S.K.; Nobles, K.; Lefkowitz, R.J. Activation-dependent conformational changes in {beta}-arrestin 2. *J. Biol. Chem.* **2004**, *279*, 55744-55753, doi:10.1074/jbc.M409785200.
109. Mandell, J.G.; Falick, A.M.; Komives, E.A. Identification of protein–protein interfaces by decreased amide proton solvent accessibility. *Proc. Natl. Acad. Sci.* **1998**, *95*, 14705-14710.
110. Mandell, J.G.; Falick, A.M.; Komives, E.A. Measurement of Amide Hydrogen Exchange by MALDI-TOF Mass Spectrometry. *Anal. Chem.* **1998**, *70*, 3987-3995, doi:10.1021/ac980553g.
111. Narayanan, S.; Mitra, G.; Muralidharan, M.; Mathew, B.; Mandal, A.K. Protein Structure–Function Correlation in Living Human Red Blood Cells Probed by Isotope Exchange-based Mass Spectrometry. *Anal. Chem.* **2015**, *87*, 11812-11818, doi:10.1021/acs.analchem.5b03217.
112. Woofter, R.T.; Maurer, M.C. Role of calcium in the conformational dynamics of factor XIII activation examined by hydrogen–deuterium exchange coupled with MALDI-TOF MS. *Arch. Biochem. Biophys.* **2011**, *512*, 87-95, doi:10.1016/j.abb.2011.05.009.
113. McDonald, C.; Li, L. Limited proteolysis combined with isotope labeling and quantitative LC-MALDI MS for monitoring protein conformational changes: a study on calcium-binding sites of cardiac Troponin C. *Anal. Chim. Acta* **2005**, *534*, 3-10, doi:10.1016/j.aca.2004.04.017.
114. Rand, K.D.; Bache, N.; Nedertoft, M.M.; Jørgensen, T.J.D. Spatially Resolved Protein Hydrogen Exchange Measured by Matrix-Assisted Laser Desorption Ionization In-Source Decay. *Anal. Chem.* **2011**, *83*, 8859-8862, doi:10.1021/ac202468v.
115. Pirrone, G.F.; Wang, H.; Canfield, N.; Chin, A.S.; Rhodes, T.A.; Makarov, A.A. Use of MALDI-MS Combined with Differential Hydrogen–Deuterium Exchange for Semiautomated Protein Global Conformational Screening. *Anal. Chem.* **2017**, *89*, 8351-8357, doi:10.1021/acs.analchem.7b01590.
120. Chen, F.; Gerber, S.; Heuser, K.; Korkhov, V.M.; Lizak, C.; Mireku, S.; Locher, K.P.; Zenobi, R. High-Mass Matrix-Assisted Laser Desorption Ionization-Mass Spectrometry of Integral Membrane Proteins and Their Complexes. *Anal. Chem.* **2013**, *85*, 3483-3488, doi:10.1021/ac4000943.

129. Köhler, M.; Neff, C.; Perez, C.; Brunner, C.; Pardon, E.; Steyaert, J.; Schneider, G.; Locher, K.P.; Zenobi, R. Binding Specificities of Nanobody•Membrane Protein Complexes Obtained from Chemical Cross-Linking and High-Mass MALDI Mass Spectrometry. *Anal. Chem.* **2018**, *90*, 5306-5313, doi:10.1021/acs.analchem.8b00236.
131. Sinz, A.; Kalkhof, S.; Ihling, C. Mapping protein interfaces by a trifunctional cross-linker combined with MALDI-TOF and ESI-FTICR mass spectrometry. *J. Am. Soc. Mass Spectrom.* **2005**, *16*, 1921-1931, doi:10.1021/jasms.8b02328.
132. Ptáčková, R.; Ječmen, T.; Novák, P.; Hudeček, J.; Stiborová, M.; Šulc, M. The application of an emerging technique for protein-protein interaction interface mapping: the combination of photo-initiated cross-linking protein nanoprobes with mass spectrometry. *Int. J. Mol. Sci.* **2014**, *15*, 9224-9241, doi:10.3390/ijms15069224.
135. Rosinke, B.; Strupat, K.; Hillenkamp, F.; Rosenbusch, J.; Dencher, N.; Krüger, U.; Galla, H.-J. Matrix-assisted laser desorption/ionization mass spectrometry (MALDI-MS) of membrane proteins and non-covalent complexes. *J. Mass Spectrom.* **1995**, *30*, 1462-1468, doi:10.1002/jms.1190301012.
137. Wortmann, A.; Pimenova, T.; Alves, S.; Zenobi, R. Investigation of the first shot phenomenon in MALDI mass spectrometry of protein complexes. *Analyst* **2007**, *132*, 199-207, doi:10.1039/B615411E.
138. Beaufour, M.; Ginguené, D.; Le Meur, R.; Castaing, B.; Cadene, M. Liquid Native MALDI Mass Spectrometry for the Detection of Protein-Protein Complexes. *J. Am. Soc. Mass Spectrom.* **2018**, *29*, 1981-1994, doi:10.1021/jasms.8b05690.
141. Bothner, B.; Dong, X.F.; Bibbs, L.; Johnson, J.E.; Siuzdak, G. Evidence of Viral Capsid Dynamics Using Limited Proteolysis and Mass Spectrometry. *J. Biol. Chem.* **1998**, *273*, 673-676, doi:10.1074/jbc.273.2.673.
